# Unjamming of particle–laden interfaces: effects of geometry and history†

**DOI:** 10.1039/d4sm01440e

**Published:** 2025-01-24

**Authors:** Carole Planchette, Gregor Plohl

**Affiliations:** a Graz Universisty of Technology, Institute of Fluid Mechanics and Heat Transfer, Inffeldgasse 25/F 8010 Graz Austria carole.planchette@tugraz.at

## Abstract

The unjamming of uniaxially compressed particle rafts triggered by the opening of a finite orifice on the opposite side is experimentally studied. Using glass beads of about 100 μm, three main behaviors are identified. Minimal unjamming does not allow significant relaxation. Axial unjamming corresponds to the growth of the unjammed domain along the compression direction with an almost constant width. The resulting channel, possibly extends through the entire raft length and may lead to partial stress relaxation. Finally, after the completion of axial unjamming, lateral unjamming may occur according to an erosion process during which jammed blocks detach from the channel edges. This is associated with important stress relaxation. By using different raft geometries, *i.e.* various raft lengths, compression levels, and opening widths, we rationalize the occurrence of these behaviors, attributing them to the rupture of the force chain network against shear and elongation, respectively. Comparing results from equally densely packed rafts prepared with three different protocols demonstrates that these two thresholds are strongly affected by the raft's history.

## Abstract 2

Compressed particle rafts are left to flow through a finite orifice placed opposite to the compressive barrier. Top view pictures evidence the influence of the opening width and raft history on raft unjamming (red) and particle release (blue).

## Graphical abstract

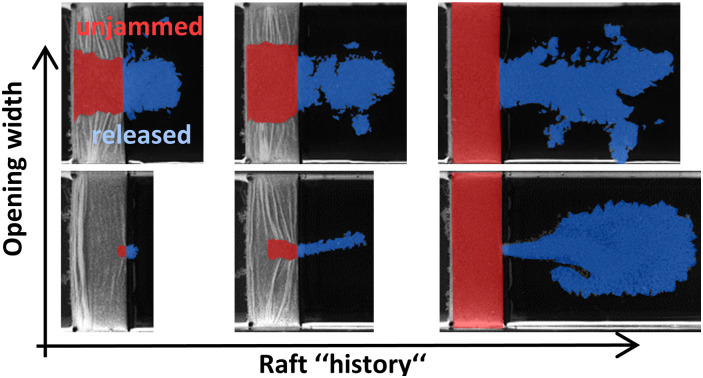

## 1 Introduction

Particle–laden interfaces have attracted interest both in the field of materials science, where they have long been used to stabilize interfaces between two fluids,^1–6^ and in the field of physics, where they serve as models for soft glassy materials.^7,8^ Similarly to insoluble surfactants, they build interfacial monolayers whose compression is accompanied at low coverage by a decrease in the effective interfacial tension, followed at higher densities by interfacial buckling.^9–12^ As for a solid beam,^13^ the selected wavelength gives access to the elastic Young's modulus (for a 3D approach) or the elastic bending modulus (for a 2D approach) of the particle–laden interface.^14,15^ Yet, in contrast to elastic sheets or molecular assemblies, particle rafts show a strong granular character associated with the development of the Janssen effect. The latter is a consequence of the non-continuous character of granular matter. Unlike in an elastic solid, in a particle assembly the stress propagates *via* discrete particle–particle contacts forming a so-called force chain network.^16^ Consequently, the mechanical properties of compressed rafts strongly depend on the particle coordination number and thus, on particle packing.^17,18^ The latter may vary over the system length^17^ or remain homogeneous.^18^ While these studies employed rafts made of monodisperse spheres, pressure isotherms measured with ellipsoidal particles confirm the importance of packing, whereas particle flipping complexifies the force chain evolution.^19^ More generally, the effect of particle packing on stress propagation is widely accepted and has been reported for diverse granular systems such as partially crystallised bidimensional disk assemblies.^20^

To date, the importance of the force chain network has mostly been demonstrated using assemblies of monodisperse spheres or disks below or around their random close packing, *i.e. ϕ*_r_ = 85%.^21^ Yet, much less is known about these systems when approaching their maximum close packing, *i.e.*
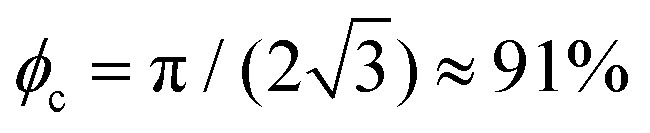
. This can be explained by the fact that, as in any self-assembling system, structures obtained *via* aggregating force – here capillary lateral attractions – require further energy possibly supplied by mechanical stirring to rearrange and thus approach crystal structures.^22^ In contrast to previously mentioned studies performed on non-equilibrated rafts, we chose here to systematically apply five compression/decompression cycles to the rafts to obtain homogeneous and dense packing close to *ϕ*_c_. This approach, inspired by inflation/deflation cycles applied to armored puddles,^23,24^ has also proven its efficiency for rafts.^25^ Working with these rafts presents several advantages. First, particle packing is well controlled and presents no inhomogeneities such as a denser front.^17^ This in turn implies that effects caused by packing variations can be eliminated, enabling the investigation of other parameters such as raft finite size.^26^ Second, such dense assemblies are expected to be highly relevant for various applications: most industrial processes involve strong strains or high rates producing packing greater than *ϕ*_r_.^27^ Foam stabilization based on arresting bubble dissolution^28–30^ is known to require very densely packed systems in which particles can only rearrange off-plane^12^ with the risk of interfacial mechanical failure and particle ejection.^31,32^ Finally, the idea to exploit supernumerary particles stored in folds to provide self-healing capacity to particle–laden interfaces^33^ implies the use of assemblies packed beyond *ϕ*_r_.

**Fig. 1:**
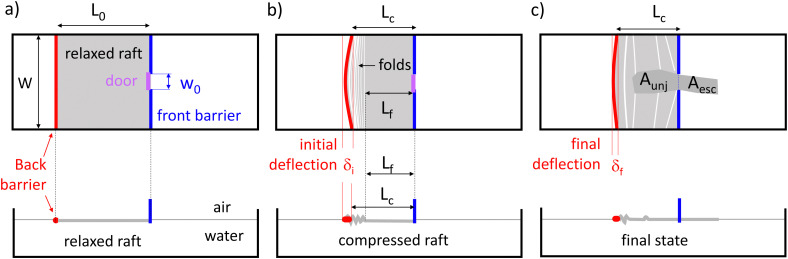
Experimental set-up from top and side views (a) before raft compression, (b) after raft compression and before local stress release, and (c) after stress release showing the final raft state. The trough width is fixed at *W* = 60 mm. The raft is compressed from the back reducing its length from *L*_0_ to *L*_c_ using a movable elastic barrier (red). Its deflection (*δ*) gives access to the lineic pressure *Π* found at the back of the raft. During compression, folds form at the back, which are separated from the front barrier by a distance *L*_f_. The door initially blocking the orifice pierced in the front barrier (width *w*_0_) is then suddenly opened and the raft evolution followed with a high speed camera placed under the trough. In its final state, the raft unjammed over an area *A*_unj_ and a surface *A*_esc_ got covered by particles passing the front barrier (blue).

In brief, despite the ubiquity of densely packed particle assemblies and their importance for practical applications, not much is known about the effect of force chain networks on their interfacial mechanical properties.^34^ Plohl *et al.*^33^ recently evidenced the importance of the compression direction on the relaxation of uni-axially compressed rafts. More precisely, the authors locally released the stress by opening a door pierced in one of the two confining barriers, as shown in Fig. 1. They showed that if the door is open at the side from which compression is applied (not shown here), the raft strongly unjams, releasing most excessive particles and achieving total stress relaxation. In contrast, if the door is open at the opposite side as in Fig. 1, only partial relaxation – if any – happens. These findings are rationalized considering the branching orientation of the force chain network. In the former case, keystone particles are located close to the opening and are removed triggering avalanches. For the latter configuration, however, keystones are at the back and the stress building up at the front is supported by arches that surround the opening. These results confirm the importance of the chain force network in triggering unjamming^35^ and in further enabling its progression within the assembly.^36^

The aim of this paper is to shed some light on the mechanical stability of the force chain network that develops in a homogeneously and densely packed particle raft. To do so, we adopt the previously mentioned test rig^33^ (see also Fig. 1) and study how back compressed rafts unjam when stress is locally released at the front barrier. By varying the raft length and its compression level, we seek to better understand how the stress is transmitted/screened. The usage of openings of various widths should provide information about typical extension of arches that may develop within the network. Of fundamental importance is also the role played by the raft history. For a given raft length, compression level and orifice width, are the mechanical properties of the force chains influenced by the method used to prepare the raft? Can this be attributed to changes in the raft cohesion, and more particularly to the strength of particle lateral attraction mediated by capillary forces?^37–40^

The used set-up, experimental protocols, and the range of studied parameters are described in Section 2 (Methods). Care is taken to independently vary the raft length, its compression level, and the opening width, so that information about stress transmission and network geometry can be deduced. Furthermore, three types of raft preparation are employed for each configuration to evaluate possible effects of raft history. The Methods section ends with a description of performed data analysis. The results, reported in Section 3, provide evidence for three types of unjamming behaviours, which are classified in regimes. Regime maps built on measured unjammed areas and raft compression levels show that unjamming is strongly modulated by both the raft preparation and the width of the opening. Three scaling laws estimating the unjammed areas for each regime confirm the validity of our classification. The paper then focuses on the transition between these regimes, which are associated with the onset of axial and lateral unjamming. The latter are interpreted in terms of network rupture against shear and elongation. Considerable variation of these thresholds with respect to raft preparation provides evidence that mixing causes a weakening of the force chain network, in agreement with simulations of jammed granular material.^36^ The consequences for raft relaxation and self-healing ability are then evaluated by linking either the residual stress or the surface getting covered by escaped particles to the unjammed area. The paper ends with the conclusions.

## 2 Methods

### 2.1 Experimental set-up

The experimental set-up – sketched in Fig. 1 and adapted from the study by Plohl *et al.*^33^ – consists of a trough to confine capillary adsorbed particles in a controlled manner. Two fixed lateral walls distant from *W* = 60 mm are combined with movable back and front barriers. The back barrier is made of an elastic rubber, whose deflection, *δ*, gives access to the local lineic pressure building at the back of the raft, see Section 2.4.2 for completeness. This is translated toward the front barrier to compress the raft at the desired level. Note that in this work the front barrier remains immobile, in contrast to some of the experiments carried out in ref. 33. The front barrier is pierced in its center by an orifice of variable width *w*_0_ equal to either 4.3, 9.5, or 19.9 mm. The door, which initially blocks the orifice can be suddenly opened to locally release the stress at the front of the raft, enabling its unjamming.

The raft evolution is observed with a high speed camera placed below the trough whose bottom plate is transparent. Typically, the frame rate is set to 3000 fps and the magnification to 173 μm per px. By analysing the recorded videos (see Section 2.3), we obtain, among others, *A*_unj_: the area that unjams, *Q*: the rate at which particles escape the initially confined domain, *A*_esc_: the final area they cover outside of the confined domain, and *Π*_i_ (*Π*_f_), the initial (final) stress measured at the raft back.

### 2.2 Experimental protocol

All rafts used in this study are made of the same particles, namely silanized glass beads. Their size distribution is Gaussian, centered at *d*_part_ = 127 μm with a narrow standard deviation of 5 μm. The contact angle with distilled water is 110° ± 5°.

#### 2.2.1 Relaxed raft production

To obtain the desired raft length, the particles are carefully weighted. Preliminary experiments have shown that for a 60 mm width trough, 0.11 g of particles are required per cm of raft. These particles are placed at the air/water interface between the fixed walls and barriers.

**Fig. 2:**
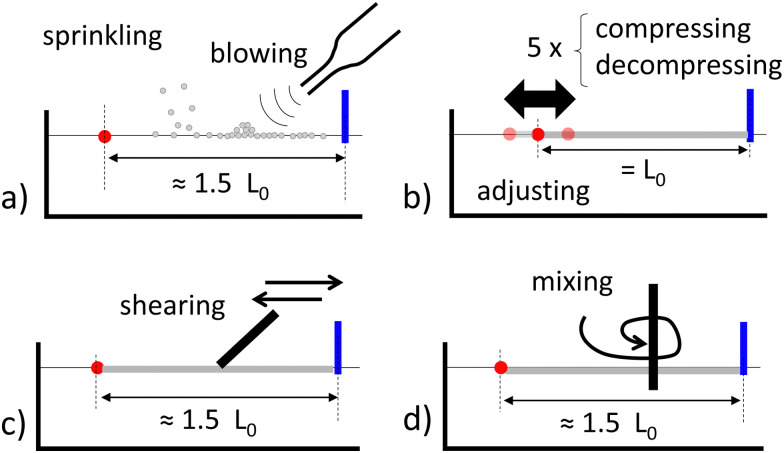
Steps involved for raft preparation. (a) Sprinkling and blowing of particles in a monolayer. (b) Raft compaction (5 cycles of quasi-static compression/decompression) and length adjustment to *L*_0_, the relaxed length. (c) Particles are sheared in the plane of the interface with the tip of a hydrophobic pipette. (d) Particles are stirred with a hydrophilic stick whose tip is immersed in the bulk water. Tempered rafts are prepared with steps (a) and (b); sheared rafts with steps (a), (c) and (b); and annealed rafts with steps (a), (d) and (b).

Rafts are then prepared according to three different protocols, which are described below. Fig. 2 shows the various steps constituting these protocols and illustrative movies can be found in the ESI.† Each protocol starts with the formation of a loose monolayer (step a) and ends with the application of five cycles of small compression/decompression at velocities in the range of 10^−3^ m s^−1^ (step b). In between, particle mixing can be applied by gently shearing (step c) or vigorously stirring (step d) the particles.

The first protocol consists of steps (a) and (b), and produces tempered rafts. Thus, weighted particles are first sprinkled at the air/distilled water interface. For all particles to accommodate in the form of a monolayer, the space between the barriers is chosen to be greater than the expected relaxed raft length. To ensure that all particles are effectively adsorbed at the air/water interface, gentle blowing is manually applied from above with a Pasteur pipette. This allows particles that may rest on top of others to roll to a free interface area. At the end of step (a), the assembly shows visible holes in its coverage, see the movies provided (ESI†). In the first protocol, step (a) is immediately followed by step (b), *i.e.* by five quasi-static small compression–decompression cycles applied by translating the back barrier. The compression/decompression are stopped when wrinkles/fractures appear. During the first cycle blowing may be used to reshape the assembly to the square confining domain. At the end of the fifth cycle, the position of the back barrier is adjusted to provide a relaxed raft, see Fig. 2 and the movies (ESI†). The length of the raft observed at the limit of back stress detection, is then measured and referred to as the “relaxed length”, *L*_0_.

The second protocol provides sheared rafts and starts similarly to the first protocol with the formation of a monoloyer (step a). Then, the particles get redistributed along the interface with the help of a hydrophobic Pasteur pipette tip, see Fig. 2(c) and movie (ESI†). Practically, the particles are locally pushed together and gaps get progressively filled. Note that the hydrophobic pipette tip never comes in direct contact with the bulk water. The process is stopped when the entire raft has been treated in this way, leaving most of the gaps closed except at the periphery. The raft preparation ends by applying five slow compression/decompression cycles and by adjusting its length to its relaxed length (step b). Note that shearing may be applied after the first compression/decompression to reshape the assembly if needed.

The third protocol is used for annealed rafts and is characterized by the strong mixing applied to the particles. After step (a), the adsorbed particles are stirred with a hydrophilic stick whose tip is immersed into the water, see step (d) in Fig. 2 and the movie (ESI†). The stirring is applied over the entire raft until all large blocks get broken into smaller ones resembling tiny islets. The resulting assembly shows the presence of holes whose dimensions are comparable to those of the tiny islets. At this stage, and as in the other protocols, five consecutive compression/decompression cycles are applied to make the raft into its relaxed state (step b).

These three protocols differ from the mixing method possibly applied to the particles between the formation of a monolayer (step a) and the final compaction of the raft to its relaxed length (step b). We chose the terms “tempered”, “sheared”, and “annealed” in reference to soft glasses assuming our particle rafts fall into this type of materials.^41^ Indeed, the possibility for athermal particles to rearrange and lower stored internal energy is directly related to the states they can explore during preparation, and thus to the applied protocol.

As mentioned, the number of particles placed at the interface is weighted and adjusted to produce rafts whose relaxed length is 40, 60 or 90 mm. In this way, we ensure that relaxed length deviations remain limited, typically less than ±1.8 mm (<5%) and indeed mostly due to weighting error and particle loss during sprinkling/blowing. Particle weighing also enables us to keep raft packing constant for all experiments. It has been measured in closed-up views and found to be 0.89 ± 0.01 for all types of preparation (data not shown). We also observe that mixing does not affect the final relaxed raft length, in agreement with the fact that the particle packing is unchanged for tempered, sheared, and annealed rafts.

#### 2.2.2 Range of parameters studied

The relaxed raft is then quasi-statically compressed by slowly translating the back barrier toward the front. The compressed length, *L*_c_, is then measured, see Fig. 1(b). For short and long rafts, *i.e.* for *L*_0_ equal to 40 mm and 90 mm, three compression levels are considered, providing values of *K* = (*L*_0_ − *L*_c_)/*L*_0_ close to 33%, 50% and 66%. For initially square rafts (*L*_0_ = 60 mm), two additional compression levels are investigated, a lower one at 25% and a higher one at 75%.

These compressed rafts are then left to unjam and relax by opening the door placed in the front barrier. By using three different front barriers, three opening widths can be investigated providing *w*_0_ equal to either 4.3, 9.5, or 19.9 mm. Each of them is used for every type of raft preparation (tempered, sheared, annealed). Together with the 11 combinations of *L*_0_ and *K*, we performed roughly 100 different experiments.

### 2.3 Data analysis

#### 2.3.1 Fold position

In its compressed state, the raft is made of a jammed and locally folded particle monolayer. The folds caused by friction undergone during compression are found at the back, thus opposite from the front barrier. They appear bright on the pictures, which allows the measurement of *L*_f_, the initial distance between the front barrier and the first fold, see Fig. 1(b).

### 2.4 Unjammed and escaped particle areas

**Fig. 3:**
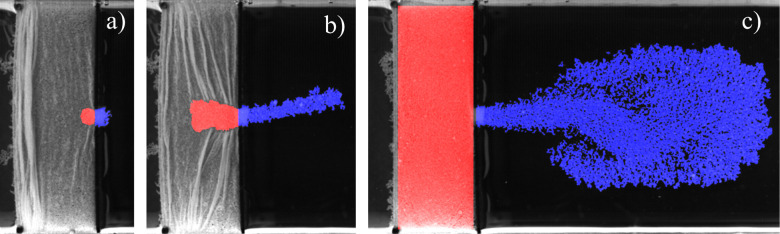
Final raft relaxation for similar geometry (*L*_0_ ≈ 40 mm, *w*_0_ = 4.3 mm and *K* ≈ 50%) with different preparations: (a) tempered, (b) sheared and (c) annealed rafts. Red color denotes *A*_unj_, the final unjammed area, and blue shows *A*_esc_, the final area covered by escaped particles. The rest is uncolored with jammed/folded areas appearing gray and uncovered domains black.

A grazing illumination makes small wrinkles visible and thus enables the visualization of the jammed area, which initially extends through the entire raft. Once the door is opened, the raft unjams – locally at least. The unjammed area contains no wrinkles and appears more homogeneous on the pictures. The unjammed particles flow through the orifice and reach the initially uncovered interface, where they are considered as “escaped” particles. Depending on the experiment, the unjamming can be almost absent, partial, or total, see also Section 3.1. The final unjammed area, *A*_unj_, is detected automatically using a machine learning plugin of ImageJ as explained in ref. 33. The final escaped area, *A*_esc_, is the surface covered by the escaped particle at the end of the experiment. It can be automatically detected using a simple threshold function on ImageJ. Examples of *A*_unj_ and *A*_esc_ detection are given in Fig. 3 for three rafts of similar geometries (*L*_0_ ≈ 40 mm and *K* ≈ 50%) obtained according to different preparation methods. From left to right, *A*_unj_ (red) and *A*_esc_ (blue) are increasing.

#### 2.4.1 Flow rates

The dynamics of escaping particles is further obtained by analysing high-speed videos. The instantaneous particle flow rate *Q*(*t*), is calculated as the temporal derivative of *A*_esc_(*t*), the surface covered by escaped particles at the instant *t*, measured from door opening. For a large number of experiments, the flow rate is found to be constant, in agreement with the reported behaviour of back compressed rafts.^33^ Thus, in the following, we will only consider constant flow rates noted *Q*. The experiments for which a constant flow rate cannot be extracted correspond to two extreme behaviours, namely very limited or total unjamming.

#### 2.4.2 Raft back pressure

Finally, we also measure *P* = *Π*_f_/*Π*_i_, the ratio between the final and initial lineic pressure at the back of the raft, see also Fig. 1. To do so, we make use of the relationship between the deflection made by the rubber band, *δ*, and the lineic pressure *Π*, which is recalled below for completeness:1
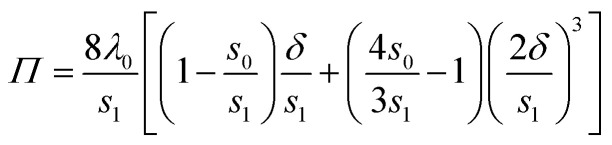
where *s*_0_ is the rubber length in the absence of stress, and *s*_1_ is the rubber length under the slight pre-stretched condition with which it is fixed to its support. The parameter *λ*_0_ corresponds to the ratio of the rubber Young's modulus and its cross section. A calibration process (not shown here) is used to precisely determine *s*_1_ and *λ*_0_ while *s*_0_ is simply measured. As the same rubber is used for all presented experiments, these three parameters are constant. A detailed derivation of eqn (1) and of the calibration process can be found in ref. 33.

Through all carried out experiments, *Π*_i_, the initial lineic pressure is found to be constant and equal to 55 mN m^−1^, in agreement with the existence of a buckling (or folding) threshold.^10,11,42^ The deflection is found to be 7.5 ± 0.5 pixels, thus with variations in the range of measurement uncertainty.

As the deflection remains small (*δ*/*s*_1_ < 2.3%), we obtain at first order:2
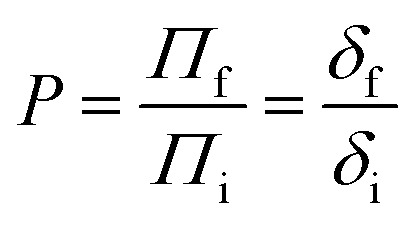
which further eliminates potential uncertainty on *s*_0_, *s*_1_ and *λ*_0_ making this method advantageous in comparison to Wilhelmy plates^26,43^ or other local force sensors.^17,18,24^

## 3 Results and discussion

### 3.1 Regimes of unjamming

#### 3.1.1 Description

Compressed rafts experience various degrees of unjamming, which are classified in three main regimes, possibly showing sub-regimes.

The first regime corresponds to the smallest degree of unjamming. The unjammed area remains limited and localized at the opening vicinity, see Fig. 3(a) or the movie in the ESI.† The rest of the raft, especially the folds found at the back, do not show any movement. No variation of the rubber deflection can be detected indicating the absence of stress relaxation. We refer to this regime as minimal unjamming and do not consider any sub-regime.

The second regime corresponds to intermediate behaviour. Unlike minimal unjamming, folds at the back get advected toward the front. The unjammed area extends in the form of a channel, whose axis aligns with the compression direction. Its width corresponds to the orifice width. Its length can extend over the entire raft or remain smaller as in Fig. 3(b). We refer to this regime as axial unjamming and further distinguish full channels, whose lengths reach the compressed raft length and partial channels, which remain shorter.

Since the previously mentioned channels extend with a constant width, further removal of jammed particles must take place according to a different mechanism. The latter resembles an erosion process during which jammed islets detach from the jammed raft into the channel. These islets instantaneously unjam and feed the channel with their particles, which flow toward the orifice, see the movies (ESI†). We refer to this regime as lateral unjamming, see also Fig. 3(c). It is important to note that lateral unjamming can only develop if axial unjamming has been completed. Furthermore, as the channel erosion can either stop before reaching the lateral walls or continue until there, we may distinguish between partial and full lateral unjamming, respectively.

#### 3.1.2 Occurrence

**Fig. 4:**
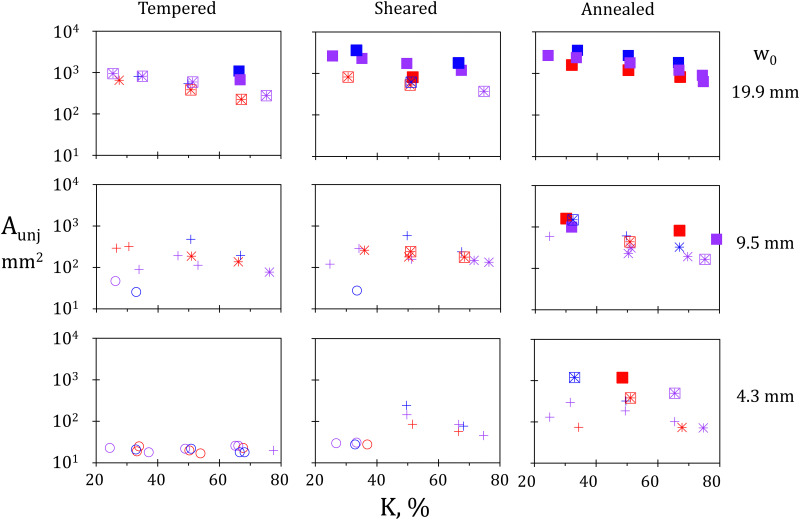
Final unjammed area in mm^2^ as a function of raft compression for different orifice widths and raft preparations. From top to bottom row, *w*_0_ is 19.9, 9.5, and 4.3 mm. From left to right column, the mixing degree increases with tempered, sheared and annealed rafts. Here and in the following, colors are used to indicate the relaxed raft length with red for *L*_0_ ≈ 40 mm, purple for 60 mm, and blue for 90 mm. Hollow circles indicate minimal unjamming; simple/double crosses indicate partial/full axial unjamming; crossed/full squares indicate partial/full lateral unjamming.

The relative occurrence of minimal, axial and lateral unjamming is represented in the form of nine regime maps, each of them corresponding to a given combination of raft preparation and orifice width, see Fig. 4. More precisely, the left column corresponds to tempered rafts, the center one to sheared rafts, and the right one to annealed rafts. Similarly, the lower row corresponds to the smallest orifice width, the center one to the intermediate width, and the upper one to the largest one. On each map, the final unjammed area, *A*_unj_, is plotted as a function of the raft compression *K* in %. The different raft sizes are indicated by different colors: red for *L*_0_ = 40 mm, purple for 60 mm, and blue for 90 mm. The regime of unjamming is coded *via* the usage of different symbols. Empty circles represent minimal unjamming; simple/double crosses show partial/full axial unjamming; and crossed/full squares indicate partial/full lateral unjamming, respectively.

The bottom left map represents tempered rafts with the smallest orifice. Whatever the relaxed raft length and compression level, and except for a single point, only minimal unjamming is observed (hollow circles). On the opposite corner, top right, the results obtained on annealed rafts with the largest opening width are reported. Full lateral unjamming always takes place, as shown by full squares. In between, intermediate behaviours corresponding to axial unjamming (crosses) are observed. This suggests that both raft preparation and orifice width influence unjamming.

Looking at the effects of raft preparation, *i.e.* comparing the various columns, we see from left to right an increase of the unjamming degree. For the smallest opening, the evolution goes from mostly minimal unjamming for tempered rafts, to only axial and lateral unjamming for annealed rafts. For intermediate orifice width, tempered rafts show coexistence of minimal and axial unjamming. For annealed rafts, minimal unjamming disappears while axial and lateral unjamming can be seen. In the case where the largest orifice is employed, minimal unjamming is not found even for tempered rafts. Instead, some axial unjamming can be observed, which disappears for sheared rafts already, leading to only lateral unjamming.

The variety of unjamming degrees can also be seen while comparing experiments performed with different orifice widths and unchanged preparation methods. Starting with the smallest orifice (bottom row) and going to the largest one (top row), a clear increase in unjamming degree can be seen. While the bottom row is dominated by minimal and axial unjamming, the top row shows mostly lateral unjamming, especially full ones. The middle row, *i.e.* the experiments carried out with *w*_0_ = 9.5 mm provides the entire variety of unjamming degrees.

Within the investigated range of mixing degrees and orifice widths, the effects produced by orifice width variations, especially the usage of the largest orifice, appear slightly more pronounced than those caused by modifying the raft preparation method. The latter are however significant, in particular for the two smaller orifices. The difficulty in characterizing and quantifying the raft mixing degree, makes these effects critical for practical applications. Indeed, our observation suggests that the unjamming – and thus relaxation and self-healing abilities of rafts – are directly related to their history, *i.e.* to a badly controlled parameter. The first quantitative evidence and interpretation of this phenomenon are presented below.

#### 3.1.3 Scaling of unjammed areas

According to our regime description, three main scalings of the final unjammed area can be proposed. When only minimal unjamming occurs, the unjammed area is expected to depend solely on the orifice width *w*_0_. Corresponding scaling provides *A*_unj_ ∝ *w*_0_^2^, and thus a first normalization:3
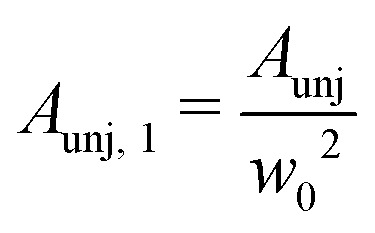
The next degree of unjamming, axial unjamming, is obtained when a channel develops. In this case, the unjammed area is expected to be proportional to the orifice width, *w*_0_, and to the compressed raft length, *L*_c_. The former roughly fixes the channel width, while the latter gives the maximum length the channel can reach. Thus, it provides a second normalization:4
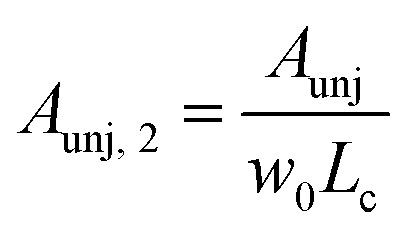
Finally, if erosion proceeds lateral unjamming occurs, which may develop within the entire raft. The scaling is given by the confined area *WL*_c_, leading to the third normalization:5
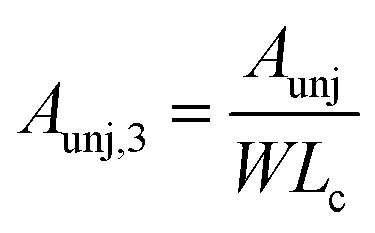


**Fig. 5:**
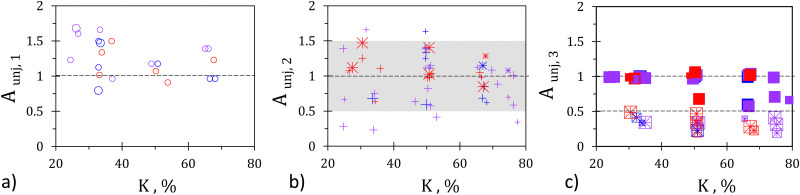
Rescaled unjammed area as a function of raft compression in %. (a) *A*_unj,1_ for minimal unjamming, (b) *A*_unj,2_ for axial unjamming, and (c) *A*_unj,3_ for lateral unjamming. Symbol colors and shapes as in Fig. 4; symbol size indicates orifice width.

In Fig. 5, data corresponding to (a) minimal, (b) axial, and (c) lateral unjamming are rescaled with previously introduced normalizations and plotted against raft compression *K*. As expected, unjammed areas measured for rafts showing minimal unjamming are satisfyingly rescaled by *w*_0_^2^, see Fig. 5(a). Indeed, all values of *A*_unj,1_ are of unity order, which is not the case if employing *A*_unj,2_ or *A*_unj,3_ (not shown).

For axial unjamming, the points are better brought together by a scaling based on orifice width and compressed raft length, see Fig. 5(b). As expected, the dispersion in channel length causes a dispersion of our data. Yet most points remain close to unity within a ±50% range (gray domain). While this may look rough, it is rather good considering the broad range *A*_unj_ covers (note the logarithmic scale in Fig. 4).

Finally, full lateral unjamming (full squares) shows an unjammed area equal to the confined area, as proven by subfigure (c) where *A*_unj,3_ is employed. A few points deviate from 1, that are systematically found slightly above 0.5. They correspond to cases where erosion reaches only one of the two lateral walls. Note that partial lateral unjamming (crossed squares), *i.e.* cases for which the erosion does not reach any lateral wall, are systematically found between 0.2 and 0.5 without any visible effect of the raft length or gate width. This can be explained by the overestimation of the unjammed area width estimated by *W* in our approach. If using *w*_0_ instead of *W*, stronger deviations are observed, which increase as *w*_0_ decreases (not shown). This indicates that despite systematic overestimation, *A*_unj,3_ provides a better scaling for partial lateral unjamming than *A*_unj,2_.

These observations confirm the relevance of using unjamming mechanisms to distinguish between three main regimes, which can in turn provide a first order estimation of the unjammed areas. At this stage, two important questions remain. First, how to predict the regime taking place for given conditions including raft history? Second, what are the consequences of unjamming on stress relaxation and self-healing ability? Answers are proposed in the next sections.

### 3.2 Regime transitions

#### 3.2.1 Channel formation: network rupture under shear

The transition between minimal and axial unjamming, *i.e.* the onset of channel formation, can be seen as the rupture of the force chain network against shear. More precisely, while the pressure transmitted from the back to the front of the raft is balanced on each side of the orifice by the front barrier reaction, this is not the case in front of the opening. There, a force *F*_o_ develops, which may overcome raft cohesion causing the extrusion of the particles found behind the orifice, and thus the channel formation. To go further, the following assumptions must be made. We consider that, at first order, the stress is fully conveyed by the folds. Beyond, a chain force network develops, which further transmits the back stress in the jammed, yet unfolded, raft part. We also assume that the distance separating the folds from the front barrier, *L*_f_, is only a function of the relaxed and compressed raft lengths. This result is experimentally verified (see Appendix A) and provides the theoretical length:

6 *L*^theo^_f_ = *L*_c_ − 0.2051(*L*_0_ − *L*_c_)

The factor 0.2051 is obtained by fitting the experimental data and may vary if different particles are used. Note, however, that it remains unchanged by the raft preparation method. Finally, we account for the Janssen effect despite the small raft aspect ratios. In agreement with results obtained in previous studies,^33,44^ we consider that total friction mobilization has been reached. This can be justified by the application of 5 cycles of light compression/decompression prior to performing the desired experiment. In contrast, the results of Saavedra *et al.*^17^ were obtained during first compression with a strong packing gradient, likely explaining the discrepancy. Thus, the force acting on the orifice width is expected to be:7
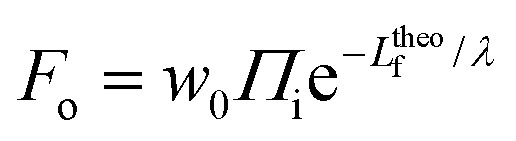
where *L*^theo^_f_ (eqn (6)) is the length on which the Janssen effect is expected to develop, *Π*_i_ is the constant initial lineic back pressure caused by compression (55 mN m^−1^), and *λ* is the screening length accounting for friction mobilization on the lateral fixed walls. The latter has been empirically obtained by fitting measured flow rates, *Q*. It is found to be 41.4 mm. For more information, please refer to Appendix B.

Within the previous assumptions, we expect the onset of channel formation to be associated with an excessive value of *F*_o_. The critical value providing the transition between minimal and axial unjamming is directly related to the raft cohesion at the orifice vicinity. It is expected to increase with the strength of the force chain network, and more particularly, with the strength of the arches embracing the orifice. Reciprocally, the critical value of *F*_o_ can be seen as an indirect measurement of the raft resistance against shear, *i.e.* of the strength of lateral interactions between the unjamming cluster and the remaining jammed domain. Note, that at first order and despite the fact that only one raft is initially present, this force might be assimilated to the lateral attraction between two separated rafts of *n*_1_ and *n*_2_ particles, given by *F*_raft_ = *n*_1_*n*_2_*F*_pair_ with *F*_pair_ the force between two single particles.^34^ As already shown, particle buoyancy cannot be neglected from the size of *O*(10^−5^) m leading here to *F*_pair_ ≈ *O*(10^−12^) N.^38,45,46^

Before probing this hypothesis, we may further try to distinguish between partial and full channels. Following previous interpretation based on a force chain network, partial channels can only be found if arches exist that can withstand the remaining back pressure. Geometrical considerations indicate that stable arches must then form angles equal or greater to the one of a cone of basis *w*_0_ and length *L*_c_. Here again, the existence of a critical value of arctan(*w*_0_/*L*_c_) can be seen as a measurement of the greatest angle made by stable arches.

**Fig. 6:**
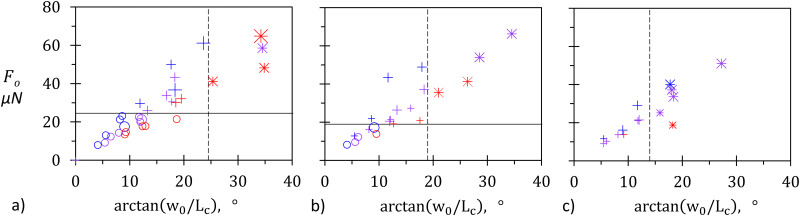
*F*_o_ as a function of arctan(*w*_0_/*L*_c_) in degrees for (a) tempered, (b) sheared, and (c) annealed rafts. Symbol colors, shapes and sizes as in Fig. 4 and 5. Continuous and dashed lines indicate the onset of axial unjamming and limit of full channel formation, respectively.

To test this interpretation, we replot the data of Fig. 4 corresponding to minimal unjamming (circles) and axial unjamming (crosses) in Fig. 6. More precisely, *F*_o_ is reported as a function arctan(*w*_0_/*L*_c_) given in degrees for (a) tempered, (b) sheared, and (c) annealed rafts. Symbol colors, shapes, and sizes remain unchanged. Tempered rafts show a clear transition between minimal and axial unjamming for *F*_o_ ≈ 24.5 μN, as marked by the horizontal black line. The vertical dashed line at arctan(*w*_0_/*L*_c_) = 24.5° separates the partial and full channels.

For sheared rafts (subfigure b), the limit of lateral unjamming is less sharp, yet clearly visible with a critical value of *F*_o_ between 12.5 and 16.5 μN (line at 14.5 μN), thus below the one of tempered rafts. The transition width could be due to the difficulty in reproducing the same level of shear and thus to start with statistically identical networks. The limit between partial and full channels is very well described by a critical angle of 19°, smaller than the one obtained for tempered rafts. These observations are in agreement with the results of Tordesillas *et al*.,^36^ which show that mixing of granular matter weakens the force chain network and facilitates its unjamming. They are also supported by the hypothesis according to which, at high packing density, unjamming is strongly affected by contact line geometry and mobility,^47^ which are themselves known to be affected by their history.^40,48^

Finally, annealed rafts (subfigure c) show axial unjamming from the smallest force *F*_o_ ≈ 9.4 μN roughly corresponding to a water/air contact line of length *d*_part_ = 127 μm and to lateral interactions between two equally sized rafts of about 3000 particles each. The data can be checked against our criterion for full channel development. As expected, a threshold marked by the dashed line at arctan(*w*_0_/*L*_c_) = 14°, *i.e.* at lower value than sheared rafts, is observed. This decrease supports the interpretation according to which the more mixed the rafts are, the less stable the chain force network is, and thus the most probable the formation of a channel extending over the whole raft length becomes.

#### 3.2.2 Erosion: inertial disintegration of elongated blocks

Lateral unjamming looks very different from the progressive axial unjamming developing along the compression direction. While the latter can be described as a regular and continuous unfolding process, lateral unjamming proceeds by successively detaching jammed islets on either side of the unjammed channel. It is important to keep in mind that after full axial unjamming, the raft is made of two separated jammed and folded blocks. While axial unjamming makes the folds move from the back toward the front, it neither causes their disappearance nor significantly modifies their orientation. Thus, the remaining jammed blocks present folds whose extremities are located at the channel edges and side walls. Assuming the particle monolayer keeps its elastic properties,^23,49^ the jammed and folded blocks are subjected to elongational stress perpendicularly to the channel axis.^25^ Raft erosion, *i.e.* the detachment of smaller jammed islets can thus be interpreted as a failure against elongation. We assume the failure to be triggered by the inertia of particles flowing in the channel. This inertia can be roughly estimated by *E*_k_ = *L*_c_*w*_0_*Φ*(*Q*/*w*_0_)^2^/2. Here *Q* is the flux of escaping particles, (*Q*/*w*_0_)^2^, their squared velocity, and *L*_c_*w*_0_, the channel surface area, which multiplied by the surface density *Φ* in kg m^−2^ provides its mass. *Φ* is given by *Φ* = 2*d*_part_*ρ*_glass_*ϕ*/3, with *ϕ*, the particle packing assumed to be constant and equal to 0.89 and *ρ*_glass_ the glass bulk density. For the erosion occurring for small blocks along the channel edge, we introduce the inertia per unit of channel length, which has the dimensions of a force:8
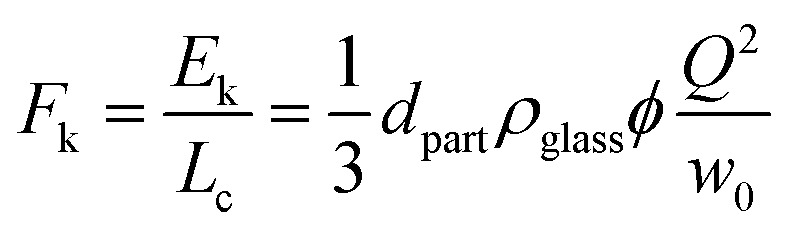


The raft cohesion, which opposes the inertial erosion, can be estimated as the product of (i) particle–particle contact density along the channel edge with (ii) the average strength of particle–particle attraction. Given the uniaxial compression applied to the raft, the lineic contact density can be estimated by *L*_0_/*L*_c_. Its excess, by reference to the relaxed state, is given by *L*_0_/*L*_c_ − 1, which can be written as *K*/(1 − *K*). The strength of the particle–particle attraction is unknown but expected to be modulated by the raft preparation. Thus, the measurement of a critical force *F*_k_ for a given value of *K*/(1 − *K*) can be seen as an indirect estimation of the raft resistance against elongation.

**Fig. 7:**
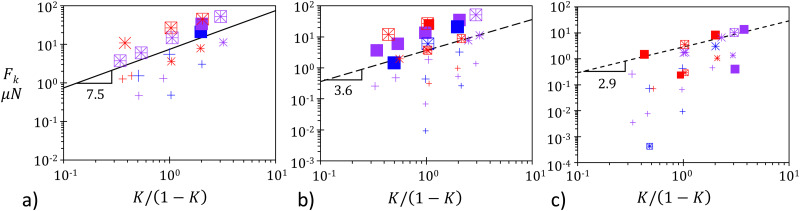
Erosion limit in the form of (*F*_k_; *K*/(1 − *K*)) diagrams for (a) tempered, (b) sheared and (c) annealed rafts. Symbol colors, shapes, and sizes are as before. The straight lines guide the eyes with slopes of 7.5, 3.6, and 2.9 μN from (a)–(c), respectively.

To probe the validity of our interpretation, all axial and lateral unjamming data points of Fig. 4 have been replotted as (*F*_k_; *K*/(1 − *K*)) diagrams in Fig. 7. To assess potential effects of raft preparation, the results are split into three subfigures, corresponding from left to right to (a) tempered, (b) sheared, and (c) annealed rafts. For tempered rafts, erosion is found above a line given by *F*_k_ = 7.5*K*/(1 − *K*). The linear variation is in agreement with the rupture of particle–particle contacts, whose density grows as *K*/(1 −*K*). The dashed line drawn for sheared rafts (b) is shifted toward lower values given by *F*_k_ = 3.6*K*/(1 − *K*) and indicates that the expected linear character of the transition is compatible with the data obtained for sheared rafts. For annealed rafts, the threshold above which only erosion occurs is further lowered to *F*_k_ = 2.9*K*/(1 − *K*). We notice that the limit is not sharp anymore with a few more erosion points found below this dashed line. They mostly correspond to the smallest orifice width equal to only 35 times the particle diameter. This questions the validity of a uniform velocity given by *Q*/*w*_0_ and thus of the erosion mechanism itself. Furthermore, we cannot exclude variations in the age or nature of the chain force network caused by the difficulties to exactly reproduce the annealing protocol. Despite these issues and the limited number of points, our explanation appears to be satisfying. Our findings clearly demonstrate that particle shearing and mixing prior to raft formation lower resistance against elongation. Our interpretation based on force chain rupture is in agreement with the data and existing literature.^36^ Finally, if compared to *F*_raft_, the lateral attraction between two rafts, *F*_k_ ≈ *O*(10^−7^) N is found to correspond to equally sized rafts of about 300 particles, *i.e.* whose typical size is 2 mm, a dimension compatible with our experiments.

### 3.3 Consequences for stress relaxation

**Fig. 8:**
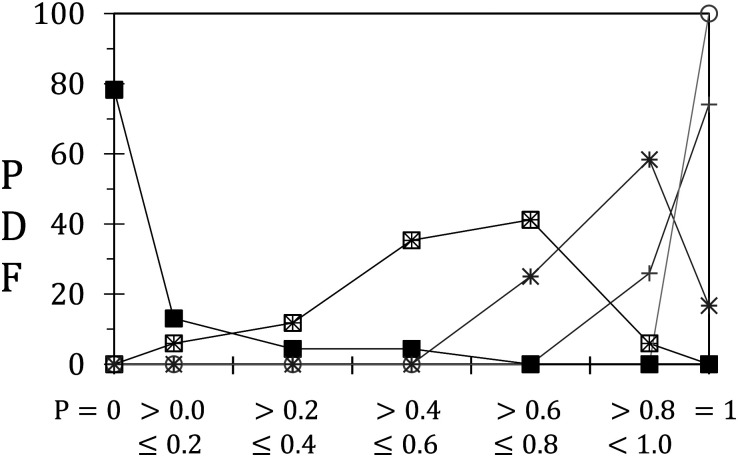
Probability distribution function (PDF) from *P*. Same symbols as before.

We now focus on the effects of unjamming on stress relaxation. To do so, we plot for each unjamming behaviour, the probability density function (PDF) of *P*, the ratio between final and initial back stress, see Fig. 8. The first and last points correspond to *P* = 0.0 and *P* = 1.0 respectively, while intermediate points are plotted for bins of width 0.2. The unjamming regimes are indicated by the same symbols as before, namely circles for minimal unjamming, crosses for axial unjamming and squares for lateral unjamming. Note that each population, *i.e.* each unjamming regime or subregime, contains at least 12 points providing an acceptable statistical meaning. The PDFs are in agreement with our qualitative interpretation: minimal unjamming does not enable any relaxation at the back, as shown by *P* = 1.0 for 100% of these 24 occurrences. Partial axial unjamming (simple crosses) leads to stress relaxation for only 26% of all events (27 points) indicating that arches often remain that fully screen the orifice. Stress decrease becomes dominant for full axial unjamming, *i.e.* for channels that develop until the raft back (>80% of the 12 measurements with *P* < 1.0). The residual back stress remains important, always greater than 60% of its initial value. This is in agreement with the existence of force chains in the two jammed blocks found on each side of the full channels. Note that the limited number of points does not allow to further probe the variations of *P* with (*W* − *w*_0_)/*W*, the portion of the raft width remaining jammed. Partial lateral unjamming (crossed squares, 17 points) marks the disappearance of *P* = 1.0. Furthermore, no case with *P* = 0.0 is observed, from which we can deduce that force chains remain on the unjammed domains that can only withstand a portion of the initially applied stress. Finally, full lateral unjamming leads to full relaxation (*P* = 0.0) for almost 80% of 23 occurrences. Other points are always found for *P* < 0.6 and typically correspond to rafts for which erosion reaches only one of the two side walls.

A strong correlation between the unjamming degree and the magnitude of back stress relaxation is evidenced. While expected, this result confirms the crucial role played by the force chain network in setting macroscopic mechanical raft properties.

### 3.4 Consequences for escaped particles

**Fig. 9:**
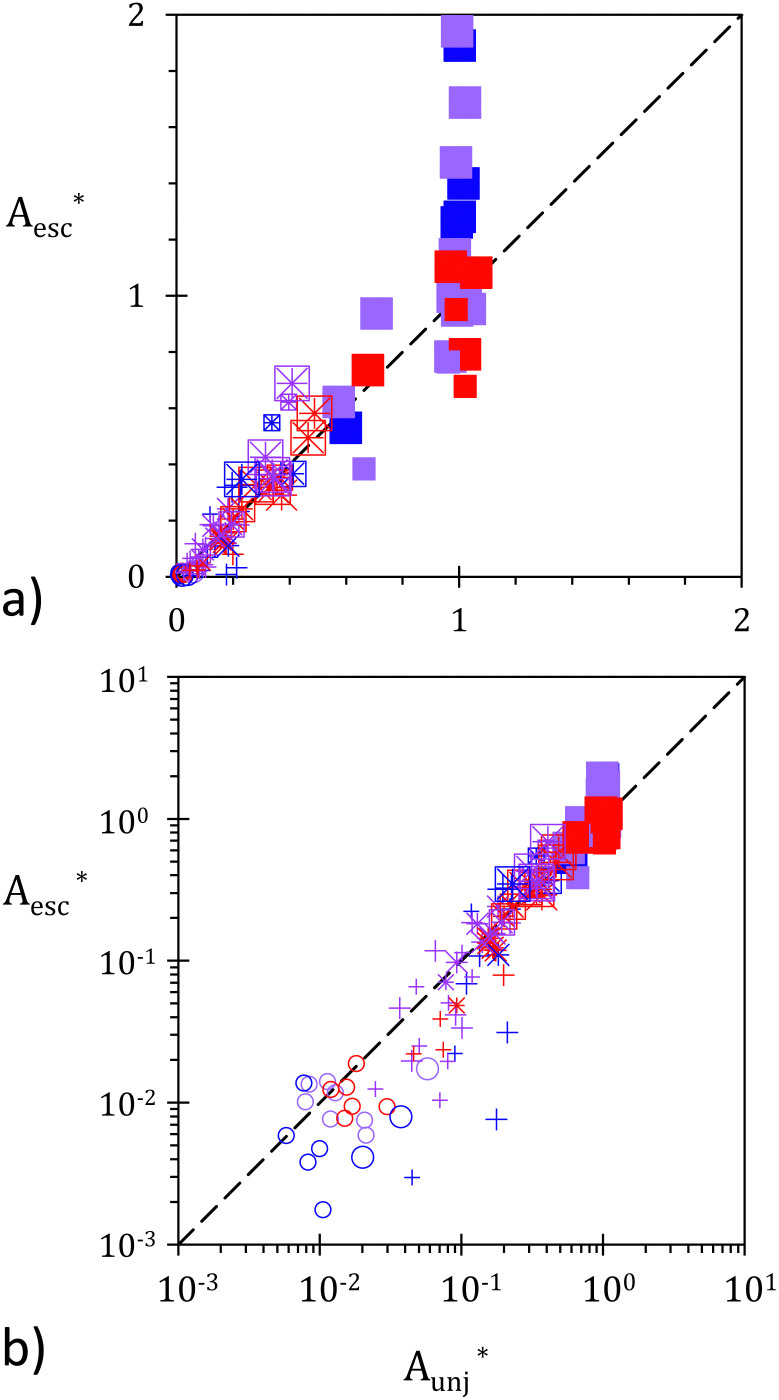
Rescaled area covered by escaped particles, 
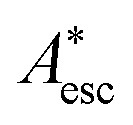
 as a function of rescaled unjammed area 
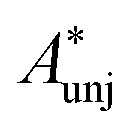
. (a) Linear scales and (b) log scales. Symbol colors, shapes, and sizes are as before.

One motivation of our study is to assess the self-healing ability of particle–laden interfaces. Capillary adsorbed particles cannot be stored in the bulk in contrast to surfactants, which can form micelles. Thus, while particles can very well stabilize interfaces of fixed or decreasing areas,^11,50^ their effectiveness becomes rather limited for extending interfaces.^44,51^ This aspect, which can be advantageously used for certain applications remains a bottleneck for other processes.^27^ By folding the particle monolayer, particle reservoirs can be obtained from which excessive particles could be released to cover opening holes.^33^ The efficiency of this strategy is related to the amount of released particles and to their migration dynamics. We focus here on the first aspect and plot 
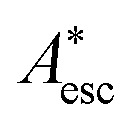
, the normalized area covered by escaped particles as a function of 
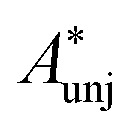
, the normalized unjammed area, see Fig. 9. The normalization of the unjammed area is made with respect to the compressed raft area, *i.e.* to *WL*_c_, providing 
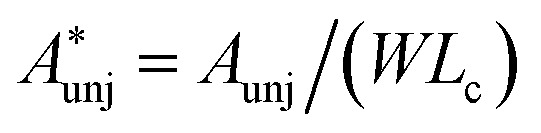
. In contrast, the escaped area is compared to *W*(*L*_0_ − *L*_c_), the area stored in the folds. Thus, we define 
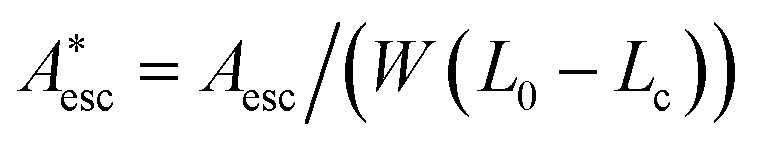
. A good correlation between these two normalized quantities can be seen. Consequently, a first reasonable estimation of the surface getting covered by escaped particles is obtained, which reads:9
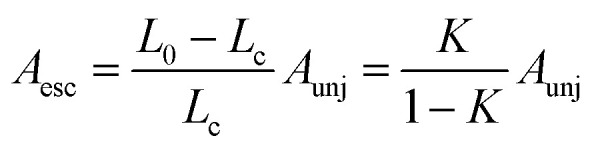


Discrepancies are mostly observed either for small values of 
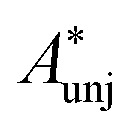
 or for large values of 
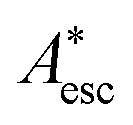
. The former can be well explained by the experimental uncertainty on *A*_unj_ for rafts which do not significantly relax. For these rafts, the contrast between unjammed and jammed domains is limited and the relative measuring error on *A*_unj_ is expected to increase. For large *A*_esc_, the deviation from prediction is caused by a change in the particle packing after escape. For rafts that fully relax, and more generally for some rafts that are subjected to erosion, the escaped particles do not form a dense assembly. With the used magnification (approx 1.5 particles per pixel), the thresholding method employed to detect the escaped particles includes voids between them, which should be excluded. Thus, while per definition the unjammed area cannot exceed the confined area used for normalization, *i.e.*
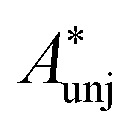
 cannot exceed 1, it is not the case of the escaped particles that may cover more than *W*(*L*_0_ − *L*_c_) as currently measured by *A*_esc_.

Yet, further accounting for the three scalings found for *A*_unj_, see eqn (3)–(5), and for the limits between these three regimes, see Fig. 6 and 7, a first reasonable prediction of raft self-healing ability has been obtained in this work. Open questions remain, which should be addressed in further investigations. One of the most critical is to unravel the mechanism(s) triggered by mixing that facilitate(s) network ruptures and thus raft unjamming. Are these effects caused by changes in the network topology such as shorter chains, less branching, or by the modification of particle–particle interactions? The latter being mediated by the deformed liquid interface,^37,52^ it is probable that raft history influences contact line at the particles.^40,48^ Different particles of various shapes and surface properties could be used to investigate this point. Ideally, the evolution of the contact line should be measured at the sub-micrometric scale, which remains a challenging task.^19,53^

## 4 Conclusions

Unjamming of back compressed rafts through orifices located at the front has been studied systematically. While the raft width (*W* = 60 mm) and particle size (*d*_part_ = 127 μm) remain unchanged, the raft length, compression level, and opening width have been varied in the following way: 40 ≤ *L*_0_ ≤ 90 mm, 25 ≤ *K* ≤ 75%, and 4.3 ≤ *w*_0_ ≤ 19.9 mm. Beyond these geometrical aspects, three raft preparation methods have been used, which differ by the degree of mixing applied to the particles but do not affect the raft packing. In this work, tempered, sheared, and annealed rafts are obtained with gentle blowing, interfacial shearing, and vigorous stirring, respectively.

Different unjamming behaviours have been observed, which could be classified in three main regimes. The transitions between them are attributed to ruptures of the force chain network and are found to be affected by the raft preparation method. The back stress relaxation and self-healing ability are governed by unjamming and increase with it.

More precisely, minimal unjamming corresponds to local and limited unjamming. Back stress never relaxes indicating that the force chain network fully screens the orifice. Yet, if the force acting at the orifice exceeds a certain value, the force chain network ruptures enabling axial unjamming. This process is characterized by the formation of a channel that extends along the compression direction with a constant width close to the orifice width. For channels that stop before the raft back, the absence of stress relaxation is observed for about 75% of the cases suggesting the presence of stable arches supporting the initial stress. Above a critical ratio of the orifice width and compressed raft length, channels systematically reach the raft back. This limit can be interpreted as the maximum angle stable arches may have. Both the critical force leading to the onset of axial unjamming and the critical aspect ratio *w*_0_/*L*_c_ are found to decrease with increasing particle mixing during preparation. The last regime, called lateral unjamming, may only develop after the completion of axial unjamming. Small jammed islets detach from the two main blocks found at either side of the channel and instantaneously unjam. This erosion process may stop before being completed or reach the side walls of the trough. In this case, the relaxation of the back stress is almost always total and most of the excessive particles initially stored in the folds are released. While we do not have yet a criterion to predict the magnitude of the erosion, its onset can be seen as the rupture of the force chain network subjected to elongation. The rupturing threshold can be derived by considering the inertial force acting along the channel edge and the density of particle–particle contacts providing the raft with its cohesion. Here again, the critical force marking the rupture threshold decreases with increasing mixing intensity of the particles during preparation.

These findings clearly indicate that mixing reduces raft cohesion facilitating unjamming and consequently increasing stress relaxation and self-healing ability. The physical mechanism(s) causing these effects may be attributed to the modification of the network topology or to modifications of particle–particle capillary lateral interactions *via*, for example, ageing of the contact lines.^47,48^ Further investigations are needed to better understand the microscopic origin of these effects.

## Author contributions

C. P: conceptualization, funding acquisition, methodology, formal analysis, and writing (original draft, review, and editing); G. P. investigation: data curation, formal analysis, and writing (review and editing).

## Data availability

The data supporting this article have been included as part of the ESI.†

## Conflicts of interest

There are no conflicts to declare.

## Supplementary Material

SM-021-D4SM01440E-s001

SM-021-D4SM01440E-s002

SM-021-D4SM01440E-s003

SM-021-D4SM01440E-s004

SM-021-D4SM01440E-s005

SM-021-D4SM01440E-s006

SM-021-D4SM01440E-s007

SM-021-D4SM01440E-s008

SM-021-D4SM01440E-s009

SM-021-D4SM01440E-s010

## Figures and Tables

**Fig. 10:**
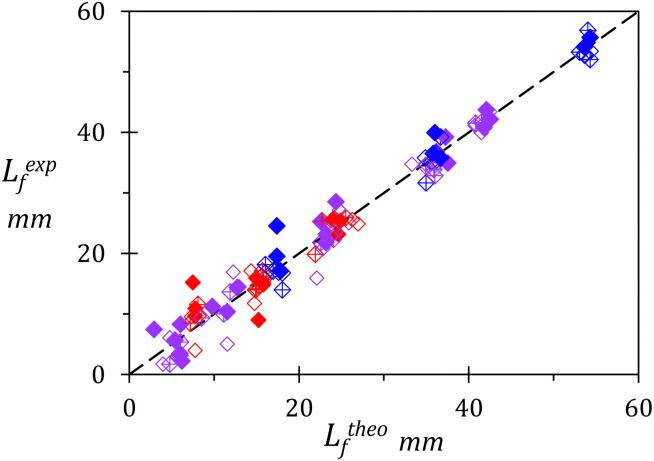
Measured *L*_f_ values as a function of *L*^theo^_f_ = *L*_c_ − *α*(*L*_0_ − *L*_c_) for *α* = 0.2051. Colors indicate the relaxed raft length with red for *L*_c_ ≈ 40 mm, purple for *L*_c_ ≈ 60 mm, and blue for *L*_c_ ≈ 90 mm. The symbols indicate raft preparation with empty/crossed/sheared diamonds for tempered/sheared/annealed rafts.

**Fig. 11:**
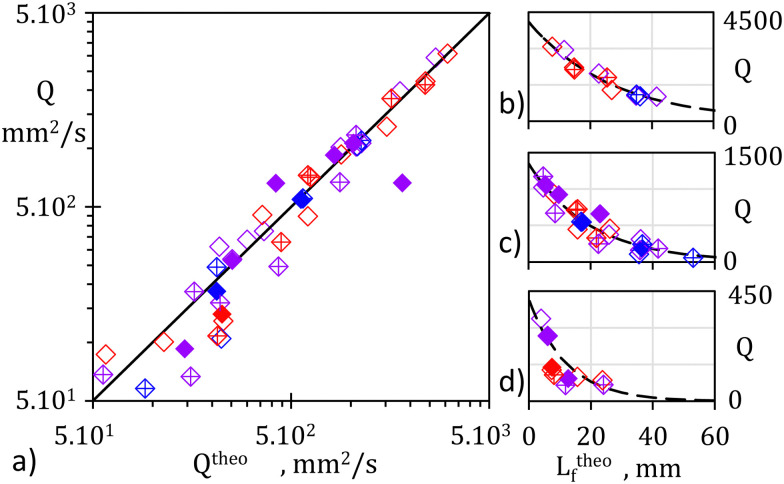
(a) Experimental *Q* values (in mm^2^ s^−1^) as a function of the prediction given by eqn (10). (b), (c) and (d) Variations of *Q* (in mm^2^ s^−1^) with *L*^theo^_f_ for *w*_0_ equal to 19.9, 9.5 and 4.3 mm, respectively. Red, purple, and blue colors for *L*_0_ of 40, 60 and 90 mm. Empty, crossed, and full diamonds for tempered, sheared, and annealed rafts, respectively.

## Appendices

### Appendices

#### Appendix A: distance between front barrier and folds

During raft compression, folds form at the back barrier, which can be easily distinguished on the recorded images. As the Janssen effect is expected to develop in the not folded section of the monolayer, it is important to measure and model the length of the not folded domain. The latter corresponds to *L*_f_, the distance separating the folds from the front barrier. The only relevant length scales are the relaxed and compressed raft lengths. We further expect the length of the folded domain to scale with the particle excess, *i.e.* with *L*_0_ − *L*_c_. Thus, a model in the form of:*L*^theo^_f_ = *L*_c_ − *α*(*L*_0_ − *L*_c_)is considered. Using the least mean squares fitting function of Matlab, we obtain *α* = 0.2051. The good agreement between the theoretical prediction and the measured values can be seen in Fig. 10, where all points have been reported, independently from employed gate width or raft preparation method. From a geometrical point of view, the factor 0.2051 indicates that the folds whose relaxed length is *L*_0_ − *L*_c_, occupy after projection in the horizontal plane 20% of this relaxed value.

#### Appendix B: flow rate modeling

For most experiments, the surface covered by escaped particles increases linearly with time indicating a constant flow rate. Note that experiments with no constant flow rates mostly correspond to minimal unjamming or to full lateral unjamming, *i.e.* to the two extreme behaviours. Experiments for which *Q* is found to be constant are used to search for a possible model. The latter does not aim to be universal but mostly to cover intermediate unjamming. Since usage of the sand-hour, granular flows are known for their steady character.^54^ In case the Janssen effect takes place, and if the flow is caused by gravity, the Beverloo law has proven its validity.^55^ Yet, in our experiments, the flow is triggered by the back lineic pressure, which is expected to be partially transmitted along the compression direction, producing a lineic pressure at the orifice. As in ref. 56 and 57, we assume the flow rate to scale linearly with the pressure at the opening. Force chain developments and friction mobilization at the walls suggest that the front pressure, *i.e.* the pressure perceived at the orifice, scales as *Π*_i_ exp(−*L*^theo^_f_/*λ*). *Π*_i_, the initial back pressure is maintained as long as folds exist. *λ*, the length-scale accounting for its progressive screening is *a priori* unknown and must be deduced from experimental measurements. To go further, another striking property of granular flows must be considered. It is their independence toward material properties.^58^ Thus, we first verify the independency of the flow rate with the raft preparation method. Comparing rafts with similar geometry, no difference can be made between the measurements obtained for tempered, sheared and annealed rafts. However, flow rates are found to vary with the orifice width (or particle size), as expected. The most common way to account for this effect has been proposed by Beverloo already^55^ and reads as (*w*_0_ − *kd*_part_)^*D*−1/2^ with *k* a numerical coefficient to be empirically adjusted and *D* the problem dimension, 2 in this work. For large orifice widths, *k* can be taken equal to 0. More complex variations have also been proposed.^59,60^ The expressions provided in ref. 55, 59 and 60 have in common to keep the dependency with the orifice width in the form of *w*_0_^*D*−1/2^, which we assume to be valid in our model. Yet, fitting experimental flow rates with *A*(*w*_0_ − *kd*_part_)^(3/2)^ exp(−*L*^theo^_f_/*λ*) shows that – whatever the value of *k* – the variations with *w*_0_ are not appropriately reproduced. Thus, we decide to fix *k* to zero and to account for orifice width effects *via* an exponential contribution in the form of exp(−*L*^theo^_f_/*w*_0_). The channel width and length being roughly given by *w*_0_ and *L*^theo^_f_, this term can *a posteriori* be interpreted as a frictional or viscous contribution. Note, nevertheless, that we do not aim for deeper interpretation here. The modeling goal is to interpret transitions between various unjamming mechanisms, which require to reproduce the experimental values of *Q*, needed for example to estimate particle velocity *Q*/*w*_0_.

Altogether, we obtain:

10

A fitting procedure run on Matlab provides *A* = 46.29 mm^0.5^ s^−1^, *λ* = 41.4 mm, and *B* = 0.2539. The agreement between the experimental values of *Q* and those of *Q*^theo^ predicted by eqn (10) is very good (correlation coefficient of 0.973), see Fig. 11(a). The subfigures b–d show *Q* as a function of *L*^theo^_f_ for decreasing orifice width (top *w*_0_ = 19.9 mm, center 9.5 and bottom 4.3). Results obtained with each width are equally well modeled, indicating that the factor exp(−*BL*^theo^_f_/*w*_0_) is appropriate here. The colors indicate the raft length and the symbols indicate the preparation method with the same convention as in Fig. 10. No systematic deviations for any of these characteristics can be seen.
